# Global norms, local institutions: a controlled comparative case study of WHO essential medicines policy adoption in Eastern Europe

**DOI:** 10.1186/s12992-026-01223-x

**Published:** 2026-06-26

**Authors:** Kristina Jenei, Elias Mossialos, Huseyin Naci

**Affiliations:** Department of Health Policy, London School of Economics and Political Science, Houghton Street, London, WC2A 2AE UK

**Keywords:** Access to essential medicines, National essential medicines lists, Policy transfer, Norm adoption, World Health Organization, Pharmaceutical governance, Eastern European health systems, Institutional legacies, International organizations, Ukraine, Moldova, Pharmaceutical policy, Universal health coverage, Health reform, Access to essential medicines, Politics, Essential medicines, Global health governance

## Abstract

**Background:**

To improve equitable access to medicines and avoid inefficient pharmaceutical spending, the World Health Organization (WHO) has long promoted five core principles regarding the selection of medicines for reimbursement: 1) the establishment of a national essential medicines list (NEML); 2) a diverse, independent selection committee; 3) an evidence-based methodology for selecting medicines; 4) integration with clinical guidelines; and 5) monitoring of use and expenditure. While many countries have formally adopted these principles, their integration into financing and procurement systems varies widely. Using Kingdon’s Multiple Streams Approach (MSA) and the policy transfer framework, this study compares how WHO selection principles were adopted in two Eastern European countries: Ukraine (higher adopter) and Moldova (lower adopter).

**Results:**

Both countries established NEMLs aligned with WHO guidance, but only Ukraine more fully embedded these principles into pharmaceutical governance. Higer adoption in Ukraine is associated with sustained donor engagement, reform momentum, and domestic leadership. In Moldova, adoption remained largely absent, associated with weak institutional capacity and episodic donor support limiting integration.

**Conclusions:**

This study illustrates how international guidance interacts with political windows, domestic will, and patterns of donor engagement to shape reform trajectories. The findings suggest that global health guidance is most influential when coupled with long-term technical support, local champions, and mechanisms for institutional continuity, helping to move reforms towards meaningful system change.

**Supplementary Information:**

The online version contains supplementary material available at https://doi.org/10.1186/s12992-026-01223-x.

## Background

Access to medicines remains one of the most urgent and inequitable challenges in global health [1, 2]. Nearly one-third of the global population lacks regular access to essential medicines, with the burden falling heaviest on those in low- and middle-income countries (LMICs), where out-of-pocket spending on medicines is often the largest household expenditure after food [3]. In some LMICs, pharmaceuticals account for up to 60% of total health spending, and roughly 90% of people must purchase medicines out of pocket [4]. Despite growing political commitments to Universal Health Coverage (UHC) progress has stalled, and financial protection has worsened in many contexts [4]. Decisions about which medicines are publicly reimbursed are now at the heart of UHC policymaking, raising difficult questions of priority-setting within constrained public budgets.

One of the most effective ways governments can promote equitable access to medicines is by developing a national essential medicines list (NEML). NEMLs guide decisions about which products are subsidized through public budgets and directly shape the availability of medicines within health facilities. When NEMLs are integrated into broader pharmaceutical governance (e.g., procurement and reimbursement processes), they can improve the efficiency of pharmaceutical expenditure and therefore improve equitable access to essential medicines. However, deciding which medicines should be included on NEMLs is a complex task as decision makers must balance scientific evidence with economic and social values in the context of scarce resources.

To support countries, the World Health Organization (WHO) developed a set of core selection principles to guide the selection processes for medicines to be included in NEMLs. These principles include: 1) the presence and use of a NEML; 2) a selection committee composed of diverse, independent experts; 3) a transparent, evidence-based methodology for selecting medicines; 4) mechanisms to link selection decisions with clinical treatment guidelines; and 5) systems for monitoring the use and expenditure of listed medicines. Over the past five decades, WHO has supported countries in adopting these principles through initiatives like the Drug Action Program (1981–2000) [5], the biennial publication of the Model List of Essential Medicines (WHO EML) and its related technical documents, and technical guidance on national implementation (2014, 2020) [6]. Since the launch of the WHO EML in 1977, the list has served as a globally endorsed reference tool, and as of 2016, over 150 countries have adopted some form of an NEML based on its contents [7].

However, a growing body of literature has documented the gap between adoption and implementation. For example, studies in low and middle income countries (LMICs) have indicated that while national lists often resemble the WHO EML, the underlying selection processes are undermined by interference from powerful elites, or lack of resources to conduct regular updates [8–17]. Importantly, divergence has been observed even among countries with comparable disease burdens and regional geography, suggesting that domestic political contexts may also shape how WHO norms are implemented [18–21].

A policy transfer lens may provide an additional lens to understand why some countries adopt international guidance, while others do not. Policy transfer refers to the movement of ideas, policies, and practices across jurisdictions, and has been widely applied to analyze global health policy reforms [22, 23], including in areas such as reproductive health [24], maternal health [25], health governance [26], infectious diseases (e.g., tuberculosis) [27], and adoption of UHC reforms [28]. However, to our knowledge, this framework has not been applied to pharmaceuticals. This is a notable gap given unique challenges medicines pose health systems. Unlike many areas where international guidance is accepted as technical standard (e.g., occupational health or treatment guidelines), medicine selection and reimbursement directly affect national budgets making them highly contested. While the same could be true for vaccines, these products are often included in global pooled procurement and financing mechanisms and may buffer them from such contestation. Whereas pharmaceuticals must be financed through national healthcare budgets; inclusion or exclusion in NEMLs therefore directly shapes both patient access and the commercial viability of pharmaceuticals within countries.

These challenges are particularly visible in Eastern European [29]. Health system reforms in the region have been constrained by path-dependent legacies such as the Semashko model [29–33]. Historical underfunding further exacerbated these constraints. For example, Ukraine spent approximately 3% of GDP on health during the 1990s and early 2000s, among the lowest levels in the WHO European Region at the time [30] (although health expenditure increased to approximately 8.2% of GDP by 2021). As a result, prescription costs were largely shifted to patients and households, with out-of-pocket expenditure accounting for 51.1% of total health spending in Ukraine [31] and 31.0% in Moldova [32]. International organizations (Ios), including WHO, promoted reforms by introducing global norms such as the principles embedded in the Model Lists. Yet, while both Ukraine and Moldova formally adopted WHO selection principles, their trajectories diverged. In Ukraine, reforms were more institutionalised, whereas in Moldova they remained intermittent and fragile. This variation raises broader questions about how international guidance is translated into national policies, and why adoption differs even among neighbouring nations facing similar constraints.

Despite these complexities of pharmaceutical reform, research in Central and Eastern Europe has largely focused on pricing and cost-containment mechanisms (e.g., managed access agreements, the introduction of health technology assessment, among other pricing mechanisms) [30, 33–42]. Far less attention has been paid to the political processes through which global pharmaceutical norms are adopted within domestic systems. This study addresses that gap through a controlled comparative analysis of two non-EU Eastern European countries with shared characteristics but differing in their adoption of WHO selection principles.

## Methods

### Conceptual definitions

This section defines the key concepts used in the analysis and explains how adoption in policy transfer was assessed. Table 1 demonstrates the operationalization of adoption levels (full, partial, none) across the five core WHO selection principles. This framework was used to assess the extent to which each principle was integrated into national pharmaceutical policy processes in Ukraine and Moldova.

**Table 1:** Conceptual definitions

WHO Selection Principles	Full Adoption	Partial Adoption	No Adoption
Presence and use of a national essential medicines list	NEML exists, is regularly updated (e.g., once in five years minimum), and directly informs procurement, reimbursement, and budgeting decisions	NEML exists but is outdated, underused, or disconnected from procurement and reimbursement systems	No NEML exists, or list exists on paper only and has no role in policy or purchasing decisions
Selection committee of diverse experts with managed COIs	Formally mandated committee with diverse, multidisciplinary membership; COI policies are in place	Committee exists but lacks consistent procedures, full independence, or enforcement of COI; membership may be narrow or politically appointed	No formal selection committee exists; decisions made ad hoc or by politically appointed individuals
Evidence-based selection process	Formal criteria for selection; structured evaluation using clinical and cost-effectiveness data	WHO EML used as a reference without formal appraisal or documentation; selection may reflect habits, or donor lists rather than evidence	No documented process for selecting medicines; no evidence review or reference to WHO recommendations
Linking selection to standard treatment guidelines	Policies supporting NEML processes mention integration with standard treatment guidelines	Some informal alignment between selected medicines and treatment guidelines, but no formal integration	No effort to align medicine selection with treatment guidelines; guidelines may not exist or are ignored
Monitoring of utilisation and expenditure	Regular, institutionalised monitoring of medicine use and spending (e.g., national procurement data and usage reports in Ukraine)	Monitoring occurs irregularly or only through donor-funded projects; not embedded in institutional workflows	No tracking of medicine use or expenditure; decisions made without utilisation data

At its core, this study investigates policy transfer, defined as “the process by which countries adopt, adapt, or reject policies from one political setting (past or present) is used in the development of policies, administrative arrangements, institutions, and ideas in another political setting” [23]. The policy transfer literature emerged in the 1990s from a subset of “policy diffusion” studies (1960s) which focused on identifying the macroeconomic conditions that facilitate or hinder policy spread. Policy transfer developed as a way to understand how and why these policies spread across contexts or time, focusing on the types of actors involved, the policy items transferred (e.g., goals, content, instruments, institutions, ideologies), motivations (e.g., dissatisfaction with existing policies, pressure from international organizations), and constraints [23]. In contrast with diffusion studies, which often leverage large quantitative datasets, policy transfer studies are often small-n, qualitative case studies exploring the reasons why one policy was adopted in one setting and not others. This theoretical approach have been widely applied across various policy domains, including health [24, 25, 27, 28], to analyze how and why certain policies are adopted, or resisted in different contexts [22].

WHO selection principles are defined as the guidelines developed by WHO to support countries in selecting medicines for national reimbursement [6]. WHO suggests countries should establish five key components: 1) an NEML (a publicly available list of priority medicines regularly updated and used to guide procurement, reimbursement, and clinical decision-making), 2) a selection committee of diverse experts (a multidisciplinary body tasked with medicine selection, with clear procedures and conflict of interest (COI) policies), 3) an evidence-based process for selecting medicines (systematic evaluation of medicines using clinical and economic criteria, ideally aligned with WHO’s evidence-informed methods), 4) mechanisms to link selection with standard treatment guidelines (alignment of selected medicines with nationally approved clinical protocols to ensure rational prescribing and use), and 5) monitoring utilization and expenditure on essential medicines (systems to track medicine use, budget impact, and compliance with national guidance). Throughout the manuscript, these components will be referred to as “WHO selection principles”. Terminology for reimbursement lists varies (e.g., national formulary, essential medicines list, or positive drug list) but these generally refer to the same policy instrument.

Policy transfer exists on a continuum, from full adoption to partial or no transfer. For analytical clarity, adoption of WHO selection principles was defined as full, partial, or absent, based on the formal presence and institutionalization of five core selection criteria.

### Theoretical approach

This study combined two theoretical approaches to explain variation in the adoption of WHO selection principles [43]: the Dolowitz and Marsh policy transfer [23] framework and Kingdon’s Multiple Streams Approach (MSA) [44]. The policy transfer framework identifies the actors involved, the type of transfer, and the motivations for transfer and constraints, helping to map the ‘who,’ ‘what,’ and proximate ‘why’ of international policy adoption. However, it does not fully explain when transfer succeeds, or why some opportunities for adoption advance while others stall. To address this, we integrated Kingdon’s MSA, which highlights how reforms depend on the alignment of problems, available solutions, and political conditions. While developed for U.S. federal policymaking, the concepts included in the MSA “have been shown to be flexible enough to be applied to nearly any place, time, or policy” [45] and has been applied to political contexts worldwide [46].

Rather than applying these frameworks in parallel, we use them in a complementary way. Policy transfer is used to identify the actors and mechanisms through which WHO principles moved into national contexts, while MSA clarifies how these agents acted as policy entrepreneurs operating within problem, policy, and politics streams. This synthesis allows us to analyse both the mechanisms of transfer (actors, types, motivations, and constraints) and the conditions of uptake (stream convergence and reform windows). In sum, policy transfer highlights the actors and ideas, while MSA explains the timing and opportunities that shaped whether these ideas were formally adopted or meaningfully institutionalised.

To integrate these approaches, we developed a policy transfer–MSA matrix, for which the results are presented within. This matrix links key dimensions of policy transfer (actors, mechanisms, motivations, content, and constraints) with the three MSA streams (problems, policies, politics). This approach enables us to situate transfer agents as policy entrepreneurs, and to analyse how WHO principles were promoted and under what conditions they gained traction or stalled. The results are presented within this matrix.

### Case selection

This study used a Most Similar Systems Design (MSSD) to explore the adoption of WHO selection principles. Drawing on John Stuart Mill’s Method of Difference, MSSD compares cases that share key structural features but differ in outcomes [47].

Case selection began with a regional approach. This choice was made under the assumption that geographic proximity may mean more similarities between countries. Indeed, the field of Area Studies in Political Science is predicated on this argument [48]. In Eastern Europe, there are nine non-EU countries classified as middle-income according to the World Bank income classification for 2019 (Table 2). These countries share common institutions, inheriting the centralized Semashko model of healthcare with state-controlled health financing models [29]. While WHO EML principles are relevant across settings, their influence is mediated differently in EU member states, where established regulatory and HTA systems structure medicine selection, compared with non-EU transition contexts where WHO guidance often plays a more direct role in institutional design.

**Table 2:** System characteristics of non-EU Eastern European countries (2019)

Country	Alignment to WHO EML (2017)	GDP per capita, PPP ($USD)	CHE (% GDP)	Pharmaceutical expenditure (% CHE)	OOP (% CHE)
EU average	**63%**	**48 194**	**9.9**	**13.6**	**15.7**
North Macedonia	No data	20 221	8.9	26	40
Ukraine	81%	14 380	7.1	26	51.1
Moldova	69%	13 413	6.5	25	31
Bosnia and Herzegovina	59%	16 428	9.0	24	29.3
Montenegro	57%	29 767	2.8	19	33.9
Albania	56%	14 791	7.00	23	56.4
Serbia	55%	21 243	8.7	NA	36.0
Belarus	No data	NA	6.0	23	25.6
Kosovo	No data	NA	NA	NA	NA

^a^Abbreviations: CHE: Current Health Expenditure; EU: European Union; GDP: Gross Domestic Product; OOP: Out of Pocket payments; PPP: Purchase Power Parity

^b^Data for alignment comes from the WHO NEML database, alignment is defined as the proportion of medicines on a national essential medicines list that are also on the WHO Model Lists; data for GDP per capita, CHE (% GDP), and OOP from World Bank; Pharmaceutical expenditure was sourced from WHO. Corruption estimates are obtained from Transparency International Corruption Perceptions Index. The index ranges from 0 to 100, with lower scores indicating higher levels of corruption and higher scores reflecting stronger transparency and governance. All data are from 2019 to ensure a more accurate comparison, as it reflects conditions before the disruptions caused by the COVID-19 pandemic and the Ukraine-Russia war

^c^All comparative health system indicators are drawn from 2019 to maintain temporal consistency across cases; later figures are referenced descriptively only and are not used for analytical comparison

Following the collapse of the Soviet Union, these countries were tasked with establishing national health systems, including pharmaceutical reimbursement mechanisms. However, challenges persist. Health systems in the region are typically underfunded with total health expenditure per capita significantly lower than EU averages. Public financing remains constrained, and out-of-pocket payments often account for 40–60% of total health expenditure [49]. These high private spending levels indicate both weak financial protection and limited public reimbursement coverage. In several transition health systems, reimbursement and procurement processes remain weakly integrated or institutionally fragmented [50]. To support this regional assessment, Table 2 provides a high-level comparison of system characteristics, including health spending, out-of-pocket payments, and alignment to the 2017 WHO EML, across non-EU Eastern European countries.

**Fig. 1:**
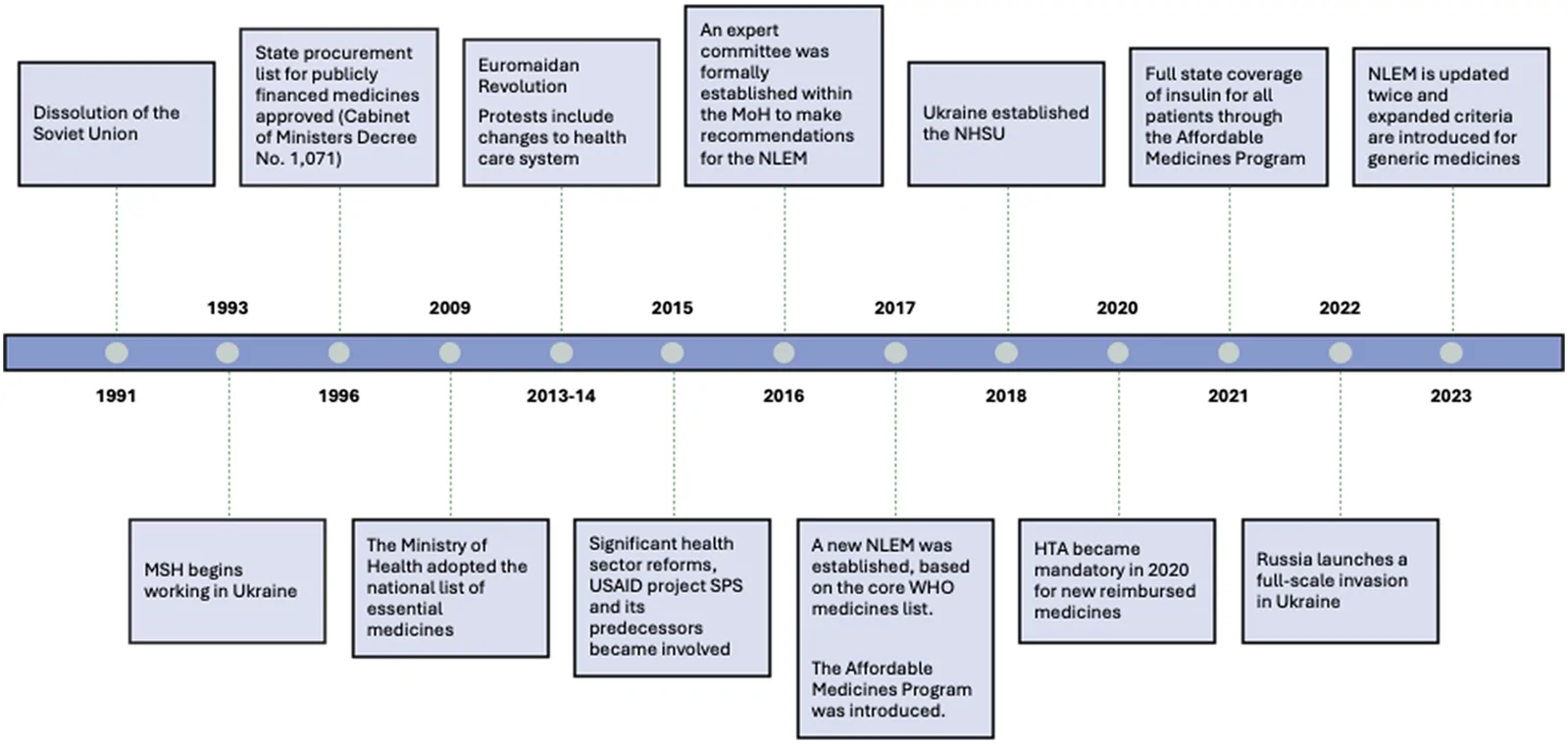
Major reforms in Ukraine. Legend: key milestones in the evolution of Ukraine’s pharmaceutical governance (1991–2021), illustrating institutional, policy, and donor-supported reforms that shaped the adoption of WHO selection principles

Next, we examined the adoption of WHO selection principles within the region by conducting an extensive document review of legislation and peer-reviewed literature. Among the possible cases, Ukraine exhibited the highest adoption across multiple selection principles. The pharmaceutical sector has undergone extensive reform since 2010 where multiple reforms integrated WHO selection principles into law and pharmaceutical governance (Fig. 1). In Ukraine, the NEML guides procurement within the public sector, is updated regularly by a committee of diverse experts, using evidence-based selection processes (including HTA). These factors made Ukraine a relatively high adaptor of WHO norms.

**Fig. 2:**
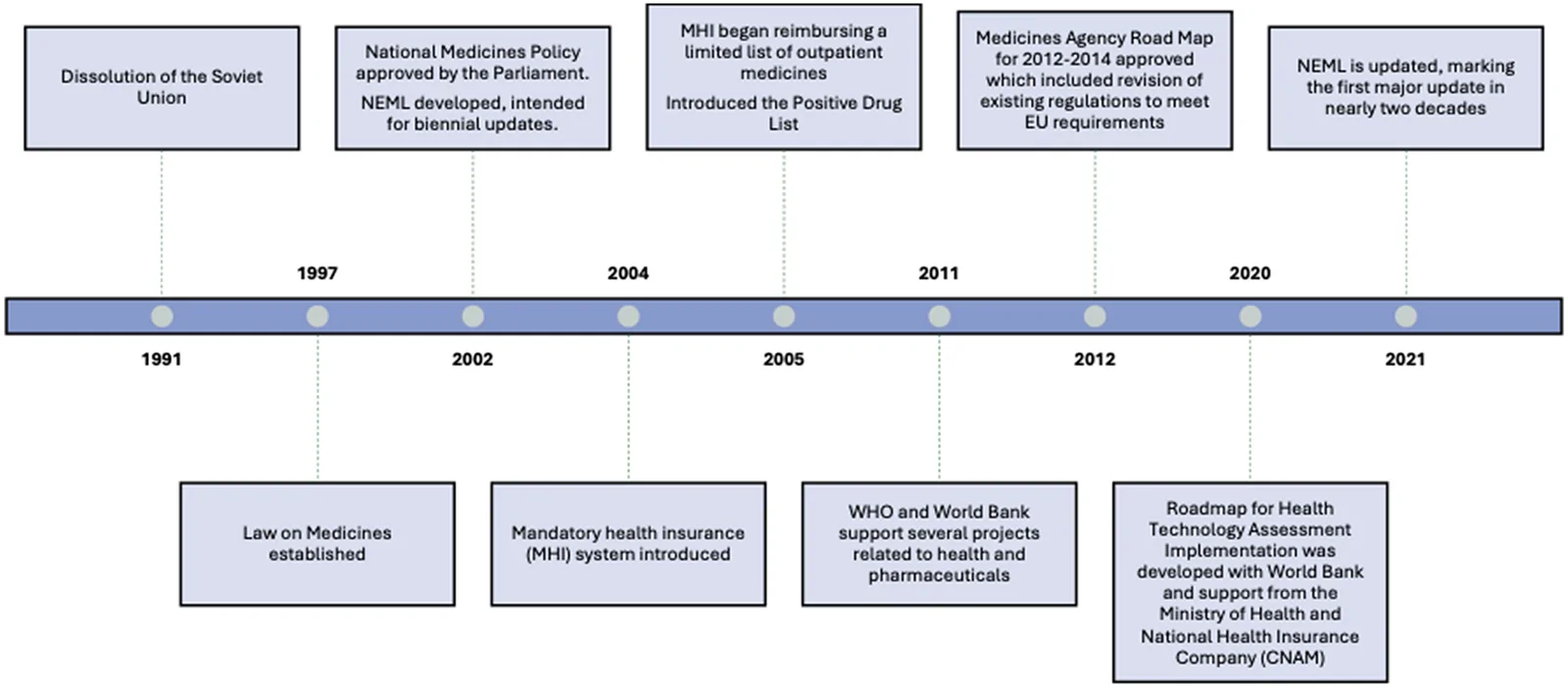
Major reforms in Moldova. Legend: key milestones in the evolution of Moldova’s pharmaceutical governance (1991–2021), illustrating institutional, policy, and donor-supported reforms that shaped the adoption of WHO selection principles

To identify a negative case, we applied Mahoney and Goertz’s Possibility Principle [51], which states that negative cases should be selected based on their potential to plausibly exhibit the outcome. Moldova was chosen because it had a plausibly high potential for reform, given its long history of donor engagement and its formal adoption of a national essential medicines list (Fig. 2). While differences among the two countries were noted (e.g., health system model, population size, cultural history, among others), Moldova shares some characteristics with Ukraine (including a border), making it a suitable comparator for controlled comparison. However, despite these enabling conditions, institutionalisation of WHO selection principles remained weaker. Partial adoption was evidenced by the presence of a national essential medicines list but the absence of regular updates, a committee of diverse experts, or consistently applied evidence-based selection processes. Nor were selection decisions linked to treatment guidelines or monitored through systematic utilisation data. This combination of plausible potential and weak institutionalisation makes Moldova an analytically valuable negative case.

### Data collection

This study drew on document and archival materials as well as key informant interviews to understand the formal design and adoption of WHO selection principles in Ukraine and Moldova. Case selection was informed by a regional mapping exercise which reviewed macro indicators (e.g., gross domestic product per capita, current health expenditure, pharmaceutical expenditure, and degree of WHO EML alignment) alongside the level of adoption of WHO selection principles. From this regional mapping, Ukraine and Moldova emerged as interesting cases.

Document and archival analysis was also used to map the policy landscape (e.g., official structures, reform timelines, donor engagement, and stated alignment with WHO guidance). This included reviewing NEMLs, legislation, selection committee guidelines, WHO and donor reports, and archival material documenting reform trajectories (e.g., SIAPS/USAID reports, early WHO technical assistance plans, and committee meeting records). Archival materials were used to contextualise early reform dynamics prior to 2000, as United Nations access rules restrict consultation of internal archival documents from the most recent 20 years. In some cases, internal documents were shared by interview participants. Most relevant were internal WHO mission reports for both countries, which outlined the pharmaceutical system and allowed triangulation of interview claims with documentary evidence. Several key documents were available in English through donor reports (e.g., USAID, World Bank), WHO publications, and peer-reviewed literature in English journals. National policy documents and websites available only in local languages were translated using Google Translate to ensure their inclusion in the analysis. We recognise that interviews alone cannot establish definitive causal explanations for reform outcomes, particularly given the small number of national stakeholders interviewed. For this reason, the document analysis was used to cross-check timelines, confirm institutional mandates, and clarify where recollections diverged from official records. To minimise error introduced by translation, we cross-checked key details against English-language secondary reports (WHO, World Bank, USAID).

### Key-informant interviews

Key informant interviews complemented the document analysis by providing insight into how WHO selection principles were understood and implemented in practice. Given the study focus on policy transfer and institutionalisation, sampling prioritised actors directly involved in the design, and implementation of selection principles, rather than those affected by downstream outcomes. This included officials within ministries of health and technical experts affiliated with international organisations engaged in pharmaceutical policy reform. Participants described as “international organisation (formerly Ministry of Health)” refer to individuals who previously held senior roles within national ministries of health and were subsequently employed by international organisations, allowing them to speak to both domestic decision-making processes and international technical assistance roles.

The predominance of internationally affiliated participants reflects the empirical reality of the reform processes within each country. In both Ukraine and Moldova, IOs played a sustained and embedded role in shaping essential medicines policy during the study period, including drafting legislation, supporting expert committees, and providing technical assistance for selection and reimbursement mechanisms. As a result, many domestic policy processes were mediated through, or closely coordinated with, international actors. Potential bias and mitigation strategies of this approach is further discussed in the limitations section.

The development of the interview guide was based on Dolowitz and Marsh’s policy transfer framework, with tailored versions for international actors and national stakeholders. Participants were identified through snowball sampling (i.e., by asking other informants about important people or organizations in the field) and prior document analysis. Interviews were semi-structured and open-ended, allowing participants to reflect freely and discuss events beyond the guide. Interviews were conducted via Zoom or Microsoft Teams, lasted between 30 and 60 minutes, and were transcribed using Otter AI or Microsoft Teams. An example of the interview guides is provided in the Supplement. Ethical approval was provided by London School of Economics and Political Science (Reference No. 407527) which deemed the study low risk. Participants affiliations were classified based on their primary role at the time of their involvement in the reforms.

### Analysis

The analysis proceeded in three steps. First, a historical timeline for each country was developed which traced key policy reforms, legislative changes, and moments of donor or WHO engagement from 1991 to 2019. This period was chosen to capture the institutionalisation of pharmaceutical governance reforms prior to major exogenous shocks, including the COVID-19 pandemic and, in Ukraine, the subsequent war, which altered procurement and decision-making arrangements. Document analysis and archival material was used to construct using document and archival sources and cross-validated with interview data.

Second, the analysis proceeded through dual application of the Dolowitz and Marsh policy transfer framework and Kingdon’s MSA. A formal deductive coding framework was developed a priori based on key constructs from these theories, including motivations for adoption, actors involved, transfer types, and barriers to adoption [48]. Interview transcripts were coded deductively using this framework, with codes refined iteratively during analysis to better reflect empirical material while remaining anchored to the theoretical constructs. Data were triangulated with documentary evidence to verify timelines, institutional mandates, and reform sequences. A comparative matrix based on the policy transfer framework was then constructed to map transfer dimensions across the two cases. Mill’s Method of Difference [47] was used to identify factors that were present in one case but not the other, helping identify factors associated with divergent trajectories

Third, insights from the policy transfer framework were integrated with Kingdon’s MSA to explain the timing and mechanisms of adoption. Interview and documentary data were re-analyzed according to the three streams of MSA (problem, policy, and politics) to assess when and how these streams converged to create policy windows (or failed to). The MSA lens provide insights into how donor influence and problem framing interacted with domestic political dynamics, while the policy transfer framework provided clarity on how external ideas were transmitted and adapted (or resisted). This combination enabled the analysis to move beyond descriptive mapping of actors and policies to analyze the interactions and institutional conditions that shaped divergent outcomes. Together, these frameworks captured both the external and internal dynamics of policy change. All qualitative data were managed and coded using a table template in Microsoft Word (Version 16.105.3).

## Results

Overall, we reviewed 238 pages of WHO archival materials and 70 peer reviewed articles or reports (Ukraine *n* = 37; Moldova *n* = 17; Eastern Europe *n* = 16). Additionally, we conducted 12 key-informant interviews with stakeholders in Ukraine (*n* = 7) and Moldova (*n* = 5) between April 1 and July 1^st^, 2025. Participants were individuals working in government (e.g., Ministry of Health or pharmaceutical regulatory agencies), as well as technical experts affiliated with international organizations (IOs) involved in pharmaceutical policy and reimbursement processes. Nearly all participants held multiple affiliations over the course of their careers (Supplement provides an overview of participant demographics).

An overview of the results is available in Table 3. Ukraine suggests convergence across all three streams. Clear problem recognition (inefficient pharmaceutical systems), credible policy solutions (WHO selection principles), and sustained political commitment (post-Maidan reform) enabled lasting reform. In contrast, Moldova suggests partial stream convergence and more limited adoption. While the problem of fragmented procurement and outdated selection criteria is recognized, and reform intent exists (problem stream), weak institutional capacity, limited donor support (policy stream) and inconsistent political windows (political stream) have inhibited full adoption.

**Table 3:** Summary of Kingdon’s MSA in Ukraine and Moldova

Policy transfer dimension	Problem stream Recognition of inefficiencies within pharmaceutical sector	Policy stream Availability & uptake of WHO principles	Politics stream Reform windows & commitment
Actors	Ukraine: Ministry of Health actors identified inefficiencies in procurement, reimbursement, and high out-of-pocket spending. Moldova: Ministry of Health and medicines agency recognised system inefficiencies, with more limited technical capacity.	Ukraine: Expert committees, WHO country office, long-term donor partners (e.g. USAID/MSH), and private sector stakeholders engaged in reform processes. Moldova: WHO country office, Ministry of Health, CNAM, and medicines agency involved, with more limited private-sector engagement.	Ukraine: Reform-oriented ministers, leadership within newly established institutions (e.g. NHSU), and donor partners. Moldova: Ministers with shorter tenures; payer agency held a strong role in decision-making.
Type of transfer	Ukraine: Reform discussions emphasised the need to move beyond inherited formulary practices. Moldova: WHO Model List served as a reference point for national list development.	Ukraine: WHO principles incorporated into formal processes, including NEML codification, HTA development, and monitoring mechanisms. Moldova: Partial incorporation of WHO principles, with weaker links between the NEML and reimbursement.	Ukraine: Reforms embedded in legislation and institutional mandates (e.g. Law on Financial Guarantees, NHSU). Moldova: Periodic NEML updates without sustained institutional integration.
Motivations	Ukraine: Public concern regarding inefficiency and corruption in pharmaceutical governance. Moldova: Formal alignment with international guidance and national concern to reform pharmaceutical policies	Ukraine: EU integration objectives, reputational considerations, donor partnerships, and market access incentives. Moldova: Compliance with donor expectations; limited commercial incentives due to market size, EU integration.	Ukraine: Post-Maidan political momentum and reform legitimacy. Moldova: Lower perceived political returns from pharmaceutical reform amid competing priorities.
Constraints	Ukraine: Industry lobbying pressures and initial enforcement challenges. Moldova: Small technical workforce and overlapping institutional responsibilities.	Ukraine: Reliance on donor funding for expert committee functioning. Moldova: Limited continuity of domestic reform champions and fragmented institutional roles.	Ukraine: Ministerial turnover affecting reform continuity. Moldova: Frequent leadership changes, payer resistance, and limited donor leverage.

Abbreviations: AmCham: American Chamber of Commerce; CNAM: National Health Insurance Company (Moldova – *Compania Naţională de Asigurări în Medicină*); EU: European Union; HTA: Health Technology Assessment; MMDA: Medicines and Medical Devices Agency (Moldova); MoH: Ministry of Health; MSH: Management Sciences for Health; NEML: National Essential Medicines List; NHSU: National Health Service of Ukraine; OOPs: Out-of-pocket payments; USAID: United States Agency for International Development; WHO: World Health Organization

### The problem stream: dysfunctional medicine selection systems and institutional fragmentation

Both Ukraine and Moldova inherited inefficient, underfunded pharmaceutical systems characterized by outdated formularies, informal payments, and disconnected planning tools such as the NEML. In both countries, inefficiencies were widely recognised. NEMLs were rarely updated, lacked formal criteria for medicine selection, and remained disconnected from reimbursement or procurement frameworks. In this sense, the problem stream was defined by a shared legacy of weak institutions.

Historically, the pharmaceutical sector in Ukraine was characterized by high out-of-pocket payments and fragmented procurement [30, 31, 52], but major reforms in 2015 enabled stronger public mechanisms for medicine selection and reimbursement. Documents indicate that efforts to rationalise pharmaceutical spending date back to 1996, when the Cabinet of Ministers approved a state procurement list for medicines financed from state and local budgets (Decree No. 1,071, 5 September 1996) [52]. This list functioned as a procurement instrument rather than a formulary in the modern sense (i.e., it was not evidence-based, was infrequently updated, and lacked explicit population health or cost-effectiveness criteria.) The first National Drug Formulary, introduced as part of a formulary-based reform programme, was published in 2009 [52, 53]. This resulted in inflated medicine prices that were often the result of entrenched corruption [52, 54, 55]. Over the following years, several reforms were attempted, such as the introduction of an outpatient reimbursement scheme for antihypertensive medicines (2012–2013), but were unsuccessful due to insufficient funding and political commitment [56]. Medicines of low clinical value were procured, while effective therapies were often unaffordable [31, 52, 57].

The 2013–2014 Maidan Revolution marked a turning point. Largescale protests transpired with public demands for improved living standards (including health system reform) which drove major changes in the health sector. By 2017, this momentum culminated in the Law on Financial Guarantees (2017), which created the National Health Service of Ukraine (NHSU) in 2018 and a public insurance plan (the Program of Medical Guarantees [58]). These changes laid the foundation for a more structured system [31, 59]. The Ministry of Health adopted a formal NEML (Ukrainian National List of Essential Medicines [NLEM]) in 2017, mandating its use for public procurement and reimbursement. The list is updated periodically, and HTA is used to inform decisions on the inclusion of new medicines, including under the Affordable Medicines Program [56]. In 2016, an expert committee with diverse, multidisciplinary membership and structured conflict-of-interest procedures was institutionalized thereafter to support NEML decisions. Evidence-based selection has been progressively formalized through HTA procedures: from 2020, all new medicines seeking reimbursement require HTA, while WHO-listed drugs with sufficient market competition are subject to expedited processes. Institutionalized monitoring mechanisms, such as the e-procurement platform Prozorro and the digital health infrastructure eZdorovya, support transparency and expenditure tracking [52, 60, 61]. Taken together, these reforms reflect a relatively high degree of institutional alignment with WHO principles on rational medicines governance within national decision-making structures.

The Moldovan pharmaceutical sector is governed by the MoH and the Medicines and Medical Devices Agency (MMDA) [33]. The NEML has historically been described as a “copy and paste” version of the WHO EML, historically lacking local adaptation or integration with reimbursement, procurement, or clinical guideline processes [34, 62]. WHO archival documents from the 2000s described it as overly broad (containing “approximately 900 drugs”), non-transparent, and based on existing prescribing habits rather than clear public health or pharmacoeconomic criteria [63]. While the list was updated in 2021, the reforms did not extend to the selection process itself, which remains opaque [33]. A new HTA mechanism under the National Health Insurance Company (CNAM) was expected to become operational in 2024 [62, 64], however, ability to strengthen evidence-based selection remains unclear [33]. Broader selection processes still rely heavily on donor funding and ad hoc expert input. Procurement responsibilities appear fragmented between CNAM and the Centre for Centralized Public Procurement in Health (CCPH), with limited use of NEML as a priority-setting tool [32].

In 2005, Moldova introduced a Positive Drug List to define outpatient reimbursement eligibility for select conditions [35]. The intention was that the NEML would identify priority medicines for inclusion on the Positive Drug List. However, the two lists remain largely uncoordinated and have limited impact on reimbursement. While several countries in the region (e.g., Albania, Bosnia and Herzegovina) share similar institutional legacies and health financing constraints, Moldova is notable for its relatively sustained engagement with WHO and other donors, including the early formal adoption of an NEML using WHO nomenclature (Lista Medicamentelor Esențiale a Republicii Moldova [65]). While Moldova has taken steps towards alignment, institutionalization of WHO selection principles remains weak. This reflects a more partial degree of transfer, in which WHO guidance was adopted formally but not embedded into financing or procurement decisions.

In sum, while Ukraine and Moldova both recognised the shortcomings of their inherited pharmaceutical systems, their responses diverged. Ukraine progressively formalised selection criteria, integrated evidence-based tools into procurement and reimbursement, and codified WHO principles into law. In contrast, Moldova maintained overlapping and fragmented systems with limited transparency and weak integration. Both countries adopted an NEML, however, only Ukraine moved towards institutionalising WHO principles, whereas Moldova’s experience suggests a more partial form of adoption.

### The policy stream: adoption of WHO selection principles

Both Ukraine and Moldova recognised the need to reform their inherited pharmaceutical systems. WHO selection principles were available globally as policy solutions, however, their adoption diverged. To understand factors associated with this divergence, we examined which stakeholders were responsible, who the key players were, and what motivated or constrained them.

Documentary evidence from WHO project memoranda from the mid-1990s corroborates interview accounts of early, embedded engagement in pharmaceutical governance reform across post-Soviet countries, including direct support to ministries of health, legislative drafting, restructuring of drug administrations, and the introduction of essential medicines–based formularies. For example, WHO Regional Office for Europe correspondence documents sustained technical assistance to ministries of health, including sector assessments, drafting of national drug legislation, restructuring of drug administrations, and support for essential medicines–based formularies across Ukraine, Moldova, and neighbouring countries [66].

In Ukraine, this long-term donor engagement was instrumental. From 1993 onward, Management Sciences for Health (MSH), funded by USAID, implemented a series of large-scale programs, along with WHO. This included the Rational Pharmaceutical Management (RPM), Strengthening Pharmaceutical Systems (SPS), Systems for Improved Access to Pharmaceuticals and Services Program (SIAPS), and SAFEMED. These projects operationalized WHO normative guidance into concrete system reforms for nearly three decades. Essential medicines were central to this agenda, as evidenced in MSH tools such as the MDS-3 [67] which was co-developed closely with WHO stakeholders.

Participants described sustained collaboration between the Ministry of Health, WHO, and long-term technical partners. As one participant noted, “we had a lot of collaboration with WHO … we were actively sharing work plans just to ensure that we are all on the same page” (participant 5, international organisation). Documentary evidence confirms that MSH supported the establishment of expert committees, including the EML committee under state programmes, and contributed to the introduction of HTA-related regulatory tools in 2019 [52, 61].

Participants also emphasised the importance of political support within the Ministry of Health for sustaining these arrangements. As one Ministry of Health official observed, “without support from the deputy minister … it’s like money was sent offshore” (participant 3, Ministry of Health). This may reflect a shared perception among reform actors that technical assistance required alignment with senior political leadership to be institutionalised.

This external support translated into tangible institutional reforms. As the national selection process closely followed WHO-recommended procedures, the more recent NEML achieved a high degree of formal alignment with the WHO Model List (approximately 90%). However, participants noted that high formal alignment did not automatically translate into contextual relevance. As one Ministry of Health official reflected, “there were a lot of products which were proposed for treatment of tropical diseases and so on, which were not so much used in Ukraine. So for two years we started to modify this list according to the requirements and demands which were needed for Ukraine” (participant 3, Ministry of Health). However, this alignment may reflect priorities beyond national burden, such as humanitarian concerns.

This observation reflects a broader tension between formal adoption of international guidance and subsequent contextual adaptation. In parallel, Ukraine established a central procurement body and a national e-procurement platform (Prozorro), strengthening transparency and coordination. However, reliance on external funding also introduced vulnerabilities. The expert committee responsible for updating the NEML ceased to operate intermittently once donor financing ended during the pre-2019 period, as no dedicated domestic budget line was established to sustain it. As one participant noted, “they just finished financing of this committee. Government did not create it in the budget … for developing this independent committee” (participant 7, international organisation). This interruption occurred prior to the subsequent institutionalisation of HTA within the State Expert Centre in 2019.

Another set of policy entrepreneurs were private sector actors such as the American Chamber of Commerce, whose motivation was to secure market access. AmCham was “very, very active” and “guided reform efforts with experts from the EU” (participant 7, IO), seeing transparency and regulatory clarity as conducive to market access. Ukraine has a large population (45.8 million in 2015) and market potential made it an attractive site for commercial influence. By contrast, several participants noted the opposite is true in Moldova: “Pharmaceutical market and economic agents are not interested to come here. So even [when] we have this list, we don’t have the medicines” (participant 10, Ministry of Health).

In comparison, Moldova experienced more episodic and project-based external engagement in medicines selection relative to Ukraine, which benefited from sustained, large-scale donor programmes over extended periods. Whereas Ukraine was the focus of long-running USAID-supported initiatives (e.g., SAPs, SIAPS, SafeMed), WHO and donor engagement in Moldova was less continuous and more concentrated around discrete reform moments.

Documentary evidence from WHO Regional Office for Europe correspondence from the mid-1990s corroborates this pattern [63, 66]. These records document WHO support to Moldova primarily through short-term missions, joint WHO–UNICEF assessments of drug management systems, and participation of senior officials in externally financed study tours and regional seminars focused on national drug policy and regulatory organisation, rather than through sustained institutional programmes. For example, For example, a WHO Assessment Mission to Moldova conducted in May 1996 focused on diagnosing systemic weaknesses in pharmaceutical governance rather than supporting the implementation of a sustained reform programme [63]. The mission, requested by the Ministry of Health, assessed drug policy, selection, regulation, quality assurance, procurement, and rational use, and identified widespread over-prescribing, weak regulatory capacity, limited use of essential medicines concepts, and the absence of a coherent national drug policy. The report recommended a broad set of reforms, including the development of an essential medicines list, strengthening of drug registration and inspection, establishment of therapeutic committees, and expanded training in rational prescribing. However, these recommendations were framed as technical guidance and follow-up assistance rather than as part of a multi-year institutionalised programme. Subsequent WHO engagement largely took the form of discrete technical missions, policy advice, and participation in regional workshops and study tours, reinforcing the episodic and advisory nature of support rather than embedding continuous implementation capacity within national institutions. WHO engagement was renewed around major NEML revisions in 2011 and 2021, with more limited involvement in the intervening years.

Participants noted that between 2015 and 2018 the Ministry of Health did not actively seek WHO assistance on medicine selection, and World Bank involvement during this period was indirect, often mediated through short-term consultancies rather than sustained institutional programmes. As a result, reform actors described selection processes as fragile and highly contingent on individual initiatives rather than institutionalised mechanisms. This fragility became visible during the COVID-19 period, when ad hoc decision-making contributed to the temporary inclusion of non-evidence-based products on the NEML, which were subsequently prohibited. These accounts highlight differences in the continuity and depth of external engagement, rather than its complete absence, and help explain divergent trajectories of institutionalisation between the two cases.

Within government, capacity was concentrated in a very small medicines department (four staff), meaning reform momentum often depended on “policy champions” at the State Secretary level. Participants highlighted that the 2021 NEML revision occurred only because one senior official personally prioritised the issue and had unusual political backing. When she resigned, the process stalled, leaving the NEML without a sustainable updating mechanism. This reliance on individual reformers (rather than enduring institutions) meant reforms were fragile and easily reversed.

Private-sector engagement was also weaker. Unlike in Ukraine, where AmCham and multinational firms lobbied for transparent rules, participants in Moldova noted that pharmaceutical companies showed little interest in their small market. As one MoH official observed, “Pharmaceutical market and economic agents are not interested to come here. So even [when] we have this list, we don’t have the medicines” (participant 10, Ministry of Health). Consequently, many medicines are on the NEML are not registered within the country and cannot be used. Where companies did engage, it was often in the form of promotion of specific products (e.g., to doctors) rather than involvement in systemic reform.

In sum, while both countries formally engaged with WHO selection principles, it seems that continuity and configuration of policy entrepreneurs were associated whether guidance was transferred. While both countries were included in early WHO support, international donors in Ukraine (USAID, MSH), WHO country and regional staff, reform leaders within the Ministry of Health and NHSU, and private actors such as AmCham and foreign pharmaceutical companies together formed a durable coalition. This sustained constellation of actors-maintained reform momentum over multiple decades, embedding WHO principles into institutional processes. By contrast, in Moldova, external support from WHO and the World Bank was more intermittent, domestic reform champions were few, and private-sector engagement was minimal. Without a stable coalition of actors, adoption of WHO selection principles remained largely symbolic and seems to have achieved the same systemic integration.

### The politics stream: reform windows and political commitment

The political context in which WHO selection principles were introduced fundamentally shaped their uptake and institutionalization. While both countries inherited fragmented pharmaceutical systems, the extent of reform depended on political windows and leadership continuity.

In Ukraine, the 2013–2014 Euromaidan protests catalyzed a policy window that elevated pharmaceutical governance to the national reform agenda. As one participant recalled, “In 2015 to 2016, after the big revolution, real reform started taking place” (participant 6, IO). When asked about this period, all participants acknowledged its significance for the pharmaceutical system. One former Ministry of Health official reflected, “It was really a period of various system reforms … focused on changes in health financing, the establishment of new institutions, and empowering those institutions with clear mandates, aligned with best practices. This demand was supported by the introduction of new regulatory tools for decision-making” (participant 2, IO).

The presence of long-standing development partners, notably USAID and MSH, meant that reform blueprints were readily available and actors seized the moment. “The best thing that can happen is when something happens politically, and you can ride that wave … you can really make change quite effectively and quickly because there’s a real desire and political will” (participant 6, IO). Their persistent engagement (what one called “beating a drum for several years” [participant 5, IO]) helped establish expert bodies, introduce HTA, and codify essential medicines principles into law despite early challenges, such as fluctuations in ministerial leadership. Here, the degree of transfer was high, with WHO principles moving beyond symbolic adoption to legal and institutional embedding.

However, the political significance of the Maidan period extended beyond the opening of a temporal policy window. Public mobilisation around corruption and state capture created sustained pressure for reform within sectors long associated with opaque decision-making, including pharmaceutical procurement. Domestic reformers within the Ministry of Health and affiliated agencies acted as policy entrepreneurs in this context, using the post-Maidan legitimacy crisis to challenge entrenched interests and reassert public authority over medicine selection and reimbursement.

International actors and WHO-aligned selection principles were actively mobilised by domestic reformers as sources of technical authority and political legitimacy in internal reform struggles. This interaction illustrates a dynamic coupling of the politics and policy streams, in which public demand and reformist leadership shaped which policy solutions gained traction, and how international norms were translated into institutional change.

Moreover, the aspiration for EU integration in Ukraine reinforced donor engagement and helped embed reforms. “Procurement reform was a first step towards integration with EU,” explained one respondent (participant 7, IO), while another noted that “the country’s readiness, its push for EU integration has been a real driver” for pharmaceutical reform (participant 6, IO). These factors acted as facilitators of policy transfer, creating external incentives and legitimacy for reform.

Moldova formally committed to harmonize its laws and regulations with the EU in 2014. However, reforms were described as more episodic and poorly institutionalized, with limited traction in the pharmaceutical sector. Frequent leadership turnover, weak institutional capacity, and inconsistent follow-up meant that EU alignment remained aspirational. One official described: “We had three Ministers of Health [in one year] … sometimes it was just one person in the medicines department” (participant 11, Ministry of Health). One Moldovan official stated: “We have only four people … responsible for all medicines, all the medical devices … physically we do not manage” (participant 10, Ministry of Health). Staff turnover meant institutional memory left with each leadership change. “The people I managed to bring into the ministry … they left together with me,” added the same official (participant 12, Ministry of Health). These constraints restricted transfer by undermining continuity and capacity.

Moldova also lacked a comparable national reform window. Although the 2014–2015 banking scandal generated public distrust, it did not catalyze health system reform. A brief opportunity arose in 2021, when a reformist government backed by parliament supported revising the NEML. “I had carte blanche to reform the NEML … there was political backing, and no opposition,” recalled one official (participant 12, Ministry of Health). However, without broader institutional embedding (e.g., streamlining legislation to support regular updates or connecting NEML with reimbursement and financing decisions), the effort faltered. “The updated list was not fully implemented, and subsequent revisions stalled” (participant 12, Ministry of Health). This represents only a partial transfer of WHO principles (i.e., formal adoption without systemic integration.

Bureaucratic resistance and political disinterest further constrained reform. Attempts to align the NEML with reimbursement policies were reportedly blocked by the national health insurer: “This was not welcomed by the payer … because that was basically moving the decision from them to the Ministry of Health” (participant 8, IO). At the same time, informal pressures from clinicians and hospitals circumvented formal processes: “We have a lot of offers from different hospitals … but they don’t present some agreements … It is very difficult not to be seen as having some economic interest” (participant 10, Ministry of Health). As a result, pharmaceutical reform remained “not on their prioritization list,” and progress became “quite stuck” (participant 11, Ministry of Health). These dynamics illustrate how entrenched interests and veto players restricted transfer. Ukraine has also experienced similar informal pressures and lobbying but has implemented structured mechanisms like HTA and transparent procurement platforms to manage these influences and strengthen evidence-based decision-making. In contrast, Moldova appears to be in an earlier stage, with a recognized lack of formal mechanisms for updating its NEML and a struggle to prevent perceived economic interests from influencing medicine selection

Finally, the geopolitical profile and smaller population reduced donor attention in Moldova. While Moldova receives substantial international assistance from different donors, including WHO, World Bank, Swiss Development Office, Global Fund, and UNICEF, resource constraints and political interest in pharmaceutical policy undermines sustainable implementation. Participants noted how WHO has had no leading role in supporting selection of essential medicines, and the government “didn’t request any support” for it (participant 9, IO). Furthermore, the differing WHO country office size and priorities are also noted, with pharmaceutical policies not being a long-standing priority for Moldova’s office: “It really depends on country office size and priorities. In Moldova for example, there was not priority pharmaceutical policies long time” (participant 7, IO). In contrast, the WHO office in Ukraine is much bigger, and pharmaceutical alignment (e.g., through the Affordable Medicines Programme) was an increasing priority [56]. Currently WHO is currently leading an HTA process focused on cancer medicines, and UNICEF will procure and donate medicines via the St. Jude platform. Despite these efforts, the overall trajectory demonstrates that in Moldova, international support has been episodic rather than embedded, which has limited full transfer.

In sum, while both countries engaged with WHO selection principles and received substantial international aid, their political trajectories diverged sharply. Ukraine’s political transition created a national imperative for reform which was associated with a relatively successful transfer of WHO principles. In Moldova, brief reformist moments were undermined by weak institutions, entrenched interests, and fragmented donor support, producing formal adoption of an NEML without embedded practice.

## Discussion

We examined how international pharmaceutical guidelines interacts with domestic adoption of WHO selection principles. Rather than offering a definitive causal explanation for divergence, this study highlights the conditions under which external norms may be more or less likely to be institutionalised. Drawing on Kingdon’s MSA and the policy transfer framework, our findings describe how different patterns of donor engagement were associated with divergent reform trajectories. In Ukraine, sustained donor support (particularly via MSH) converged with political will and reform momentum after the Euromaidan Revolution. This alignment enabled WHO selection principles to become embedded within broader health system reforms, including their integration into HTA processes and links to financing and reimbursement. By contrast, adoption in Moldova was weakly institutionalised. A brief reform window in 2021 enabled revision of the NEML, but the lack of continuity in political leadership, limited human resources, and intermittent donor alignment closed the politics stream before deeper institutionalisation could occur.

A central insight from this study is that country alignment with international guidelines does not guarantee implementation or meaningful systems change. Adoption is rarely a linear process; rather, policies pass through institutional capacity. Heinrich (2021) describes this process as being “transformed by their journey through professional communities, and through time.” [68] This is especially relevant in the area of pharmaceuticals which is a domain uniquely vulnerable to intense lobbying and requiring robust institutional mechanisms to ensure evidence-based processes. Indeed, studies on the adaptation of the WHO EML in countries reveal a more nuanced, less linear process of policy transfer, highlighting negotiated uptake and the profound influence of donor relationships and existing systems [10, 28]. Our findings suggest that IO guidance can be used strategically by domestic actors to navigate internal political economy challenges. In Ukraine, WHO selection principles and donor-backed committees provided reformers with legitimacy to resist entrenched interests and to structure pharmaceutical decision-making transparently, even in a context of strong lobbying. Professional communities (e.g., expert committee members, HTA specialists, reform-oriented MoH and NHSU officials, and associations such as AmCham) further reinforced the adoption of WHO principles by promoting evidence-based and transparent processes. In Moldova, by contrast, the limited presence of commercial lobbying (e.g., large pharmaceutical companies and vested American interests) meant there was less external pressure to institutionalise reforms. Overall, our findings indicate that closer attention to how IO guidance is strategically deployed by domestic actors can complement existing accounts on public sector reform processes. Domestic integration (rather than international transfer such as the case with this study) will be an important area for further research.

Second, domestic political leadership emerged as a central mediating factor in the adoption and institutionalisation of WHO selection principles. In Ukraine, reform-oriented ministers, senior Ministry of Health officials, and civic society leadership within newly created institutions such as the NHSU acted as domestic policy entrepreneurs who translated public demand for anti-corruption and transparency into concrete institutional reforms. These actors did not simply implement externally designed solutions, but legitimised international norms to advance domestic reform agendas and counter entrenched interests within the pharmaceutical sector. By contrast, in Moldova, leadership dynamics were characterised by short ministerial tenures, limited bureaucratic depth, and a heavy reliance on individual champions rather than institutionalised decision-making structures. Although reformist leaders temporarily advanced NEML revisions, the absence of stable leadership coalitions and protected institutional mandates constrained sustained adoption. These contrasts highlight how domestic leadership operates at the intersection of the politics and policy streams, shaping whether international guidance becomes embedded within national decision-making or remains largely symbolic. In practice, leadership was closely tied to the presence or absence of domestically embedded expert capacity, which determined whether reforms could be operationalised beyond formal adoption.

Third, this study underscores the caution in using alignment indicators for assessing access to essential medicines. A substantial body of literature has examined how closely countries align with WHO, either by comparing NEMLs with the WHO EML [18–20, 69–74], or by assessing the extent to which national selection procedures follow WHO-recommended principles across LMICs (e.g., Mali [75], Pakistan [11], Tanzania [12], Uganda [76], Nepal [15], South Africa [77], Brazil [16], Ghana [76], Kenya [8, 17], and Mexico [9]). These studies have been valuable in documenting patterns of technical compliance and have increasingly been used as proxies for progress in pharmaceutical governance, including within the WHO Roadmap for Action on Health Products and Technologies 2025–2030 [78]. However, this emphasis on formal alignment risks obscuring the conditions required for implementation. Evidence from this study may suggest that adopting WHO principles can function as a signalling device to demonstrate compliance with international norms, without necessarily reshaping domestic decision-making processes. In both Ukraine and Moldova, participants described how formal adoption helped secure legitimacy with external partners even when institutional integration remained partial or contested. By shifting attention from alignment outcomes to adoption processes, this study contributes to a more nuanced understanding of how international norms are mobilised within national pharmaceutical governance.

A critical reflection is also warranted. While WHO selection principles are widely regarded as global best practice, their alignment may not always translate into better outcomes for population health. High adoption can risk creating dependency on external models and encourage “one-size-fits-all” approaches that overlook local priorities, fiscal realities, or implementation capacity. As Andrews, Pritchett, and Woolcock (2017) caution, such “isomorphic mimicry” can produce reforms that appear aligned on paper but remain functionally hollow [79]. The findings may suggest examples of this given instances in both countries where formal adoption of an NEML did not translate into systemic change nor implementation of other selection principles. Even in Ukraine, reforms tied to donor cycles raised questions about how deeply institutionalised these processes will remain once external funding declines (e.g., the lack of funding for the Expert Committee). These examples suggest that alignment with WHO norms, while valuable for legitimacy and signalling, is insufficient on its own and may even divert attention from building context-specific mechanisms that can deliver durable improvements in access to medicines [79]. However, Ukraine’s trajectory also demonstrates that mimicry is not an endpoint and that under the right conditions (e.g., political windows, and strong domestic leadership) symbolic adoption can transition into more functional reform.

Finally, a key contribution of this study lies in highlighting how deeply embedded institutional legacies may shape the adoption of IO guidance. In Ukraine, reforms were layered into existing structures through the retention of a tax-based system and the creation of a strong institutional anchor in the National Health Service. Moldova, by contrast, followed a social health insurance model but faced fiscal constraints and demographic decline through Romanian emigration, which weakened its capacity to institutionalise WHO principles beyond formal listing. Even the shift to social health insurance across Eastern Europe was often driven by an “anti-communist backlash,” where domestic actors perceived financing models associated with the communist regime as unacceptable, even when recommended by prominent IOs [80]. Our findings may suggest under certain conditions (e.g., sustained donor engagement, mobilised domestic actors, and the opening of a major reform window), international norms can be more fully embedded, even within post-communist institutional frameworks, as illustrated by Ukraine. This aligns with previous research noting that Ukraine has at times been comparatively more receptive to guidance from organisations such as WHO, particularly in policy areas perceived as less politically sensitive and not tied to financial conditionality [81]. Our study adds to this literature by indicating that, even in the politically and economically contentious domain of pharmaceutical policy, such deeper embedding may be possible.

There are several limitations that should be acknowledged. First, controlled comparisons using Mill’s Methods are subject to two major shortcomings: over-determination and sampling bias [48]. Over-determination occurs when there are multiple causes that could explain the outcome, therefore obscuring the factors that were most important. To mitigate these concerns, we followed Lijphart’s (1971) recommendations, choosing Ukraine and Moldova based on comparable structural characteristics but divergent reform trajectories [82].

Second, the interview sample included a high proportion of participants with current or prior affiliations to international organisations. This reflects the empirical reality that international actors played a sustained and embedded role in pharmaceutical governance reforms in both Ukraine and Moldova during the study period. However, this composition may privilege international perspectives on reform processes and attribution of influence. The analysis therefore does not treat interview accounts as neutral. Interview data were triangulated with national legislation, archival WHO and donor materials, and documented reform timelines to reduce reliance on retrospective or self-reinforcing narratives. Perspectives from domestic clinical associations, civil society organisations, and parliamentary actors were less represented, and future research could usefully examine how these groups interpret and contest essential medicines reforms.

Third, interviews may be subject to recall bias given the historical nature of events, and our sample (12 participants, seven of whom were affiliated with international organisations) may have skewed perspectives toward donor priorities. However, this composition reflects the dominance of international actors in essential medicines policy during the study period. We sought to mitigate these risks through triangulation with national legislation, policy documents, and archival sources. Finally, some policy documents were only available in Ukrainian or Romanian. Although Google Translate and bilingual reports were used, and most key documents were available in English, minor inaccuracies in terminology cannot be ruled out.

## Conclusion

This controlled comparative case study examined the factors associated with the transfer and timing of WHO selection principles in Ukraine and Moldova. Both countries experienced similar challenges, including non-transparent decision-making, weak links between the NEML and reimbursement mechanisms, and fragile governance of essential medicines policies. While both formally adopted aspects of WHO selection principles, Ukraine institutionalised them more extensively through reforms supported by sustained donor engagement and strong domestic leadership. In Moldova, adoption seems to have been intermittent with limited institutional integration.

Our findings suggest three broader insights. First, international norms such as WHO selection principles may be embedded institutional frameworks, but this appears more likely when domestic political windows converge with domestic leadership and with long-term donor partnerships. Second, reform momentum is not necessarily self-sustaining. Moldova illustrates how partial uptake, even when initiated by reformist actors, may falter without institutional continuity or resource commitments. Third, international guidance should be understood as a process that depends on political timing, local ownership, and the interplay between domestic and international actors, rather than on simple measures of policy alignment.

By analysing policy transfer processes in relation to Kingdon’s MSA, this study contributes to the literature on global health governance in transition settings (e.g., LMICs). We suggest that there may be value in pairing global policy tools (e.g., WHO EML) with sustained, context-sensitive support for institution-building, especially in settings where capacity constraints may hinder long-term changes. For policymakers, our findings suggest that aligning formally with WHO guidance is insufficient without institutional continuity for implementation. Countries may anticipate donor cycles and embed mechanisms that can survive leadership turnover if reforms are to translate into durable improvements in access to essential medicines [83].

## Electronic supplementary material

Below is the link to the electronic supplementary material.

Supplementary Material 1

## Data Availability

The datasets generated and analyzed during the current study are not publicly available due to confidentiality agreements with interview participants.

## Abbreviations

CCPH: Centre for centralized public procurement in health
CNAM: National health insurance company
COI: Conflict of interest
EU: European Union
IO: International organization
MMDA: Medicines and medical devices agency
MSA: (Kingdon’s) multiple streams approach
MSH: Management sciences for health
MSSD: Most similar systems design
NEML: National essential medicines list
NHSU: National health service of Ukraine
RPM: Rational pharmaceutical management
SPS: Strengthening pharmaceutical systems
UHC: Universal health coverage
WHO: World health organization
EML: (World Health Organization) model lists of essential medicines
