# Supplementary material for: Global norms, local institutions: a controlled comparative case study of WHO essential medicines policy adoption in Eastern Europe
Source: Global Health. 2026 Jun 26;22:65. doi: 10.1186/s12992-026-01223-x (PMC13563743; doi:10.1186/s12992-026-01223-x)
Supplement: Supplementary file 1 [file 12992_2026_1223_MOESM1_ESM.docx]

**Appendix**

**Appendix 1**

**Table S.1 Operationalisation of the adoption of WHO selection principles**

| **WHO Selection Principle** | **Full Adoption** | **Partial Adoption** | **No Adoption** |
| --- | --- | --- | --- |
| **Presence and use of a national essential medicines list** | NEML exists, is regularly updated, and directly informs procurement, reimbursement, and budgeting decisions | NEML exists but is outdated, underused, or disconnected from procurement and reimbursement systems | No NEML exists, or list exists on paper only and has no role in policy or purchasing decisions |
| **Selection committee of diverse experts with managed COIs** | Formally mandated committee with diverse, multidisciplinary membership; COI policies are in place and enforced | Committee exists but lacks consistent procedures, full independence, or enforcement of COI; membership may be narrow or politically appointed | No formal selection committee exists; decisions made ad hoc or by politically appointed individuals |
| **Evidence-based selection process** | Formal criteria for selection; structured evaluation using clinical and cost-effectiveness data | WHO EML used as a reference without formal appraisal or documentation; selection may reflect habits, or donor lists rather than evidence | No documented process for selecting medicines; no evidence review or reference to WHO recommendations |
| **Linking selection to standard treatment guidelines** | Reimbursement decisions and treatment guidelines are harmonised; medicines on the NEML are directly reflected in clinical protocols | Some informal alignment between selected medicines and treatment guidelines, but no formal integration | No effort to align medicine selection with treatment guidelines; guidelines may not exist or are ignored |
| **Monitoring of utilisation and expenditure** | Regular, institutionalised monitoring of medicine use and spending (e.g., national procurement data and usage reports in Ukraine) | Monitoring occurs irregularly or only through donor-funded projects; not embedded in institutional workflows | No tracking of medicine use or expenditure; decisions made without utilisation data |

**Appendix 2: Semi-structured interview guides**

**Interview guide for government officials**

Section 1: Introduction to study motivation and consent

1. Introduction to the study
2. Consent

Section 2: Understanding the pharmaceutical reimbursement system

3. Can you describe how the reimbursement system for medicines works in your country?
   a. Who is responsible for deciding which medicines are reimbursed?
   b. What are the main criteria used for making these decisions?
4. How does a medicine get added to the reimbursement list?
   a. How often is the list updated?
5. What role does the reimbursement list play in medicine access?
   a. Does it apply to both outpatient and inpatient care?

Section 3: Variability in policy transfer and international influence

6. What role have international organisations played in shaping pharmaceutical reimbursement policies?
   a. Are there any examples where WHO guidance directly influenced national policies?
7. What motivated your government to adopt WHO essential medicines policies?

IF NOT adopted: Some countries have adopted the essential medicines policies, in your opinion, why didn’t your country follow the same route?

- Where there attempts? What alternative policies are put in place instead?

8. What were biggest barriers to adopting WHO essential medicines policies?
9. How would you assess the success of WHO policy adoption in your country?
   a. Have the changes improved medicine access or system efficiency?
   b. Are there any lessons learned that could inform future policy decisions?
10. Anything else you think I should know?
11. Anyone else I should talk to?
12. Do you mind if I contact you later if I have further questions?

**Interview guide for international organisations**

Section 1: Introduction to study motivation and consent

1. Introduction to the study
2. Consent

Section 2: Understanding involvement in case countries

3. Can you describe your organisations involvement in pharmaceutical policy in [Ukraine/Moldova]?
   a. What types of support have been provided (e.g., technical assistance, funding, regulatory guidance)?
   b. Who were the key actors during this time?
4. How did the essential medicines concept become part of policy discussions in these countries?
   c. Where did it originate from?

Section 3: Understanding policy transfer variability

5. Some countries (e.g., Ukraine) has fully adopted WHO essential medicines policies, while others (e.g., Moldova) has not. Why do you think this difference exists?
6. What factors determined whether a country adopted this guidance?
   d. Conditions on funding? Key stakeholders?

Section 4: Barriers and challenges to policy transfer

7. What are the biggest barriers to adopting WHO essential medicines policies in this region?
8. From your perspective, how successful has WHO essential medicines policy adoption been in these countries?
   e. Have the policies led to measurable changes in medicine access or system efficiency?
   f. Are there lessons learned that could improve efforts in similar contexts?
9. Anything else you think I should know?
10. Anyone else I should talk to?
11. Do you mind if I contact you later if I have further questions?

| **Table 3. Overview of the pharmaceutical policy systems** | | |
| --- | --- | --- |
| **Country** | **Ukraine** | **Republic of Moldova** |
| **Governance** | - Ministry of Health of Ukraine oversees national health policy - State Expert Center (MoH) grants marketing authorisation. - State Expert Center (MoH) conducts HTA - HTA findings inform MoH decisions. - MoH approves recommendations - National Health Service of Ukraine (NHSU) implements the reimbursement system and contracts pharmacies under the Affordable Medicines Program - Medical Procurement of Ukraine handles centralised procurement | - MoH overseas national health policy - AMDM approves medicines - Permanent Commission of the MoH makes decisions regarding the NEML. - Council for Reimbursed Medicines (interdepartmental organisation) makes decisions about reimbursed medicines - CNAM (autonomous state organisation) manages the health insurance system and is responsible for the reimbursement of medicines within the framework of mandatory health insurance. - Pharmacotherapeutic Committees within health care facilities make decisions on procurement and use of medicines - Pharmacies distribute reimbursed medicines according to co-pays (receive compensation from CNAM). |
| **NHS/SHI** | NHS | SHI |
| **Funding of public health services** | - Predominantly tax based funding | - SHI contributions and tax transfer from central government - State subsidies for certain groups - Co-payments and out-of-pocket spending |
| **Public health insurance established** | 2018* | 2004^1^ |
| **Marketing authorisation** | - Ministry of Health | - Medicines and Medical Devices Agency |
| **Name of reimbursement list(s)** | - National list of essential medicines (NLEM) “Affordable medicines programme”. - Medicines for centralised procurement programs | - The Reimbursed List of Medicines with INNs (167 INNs in outpatient) - The Reimbursement List of Medicines and Medical Devices with Trade Names (brand name products) (outpatient?) - National hospital formulary |
| **NEML established** | 2017 | - Moldova started to implement the EML and promote rational use in public and private sector since the mid1990s^2^ - Officially established (2002) |
| **Role of the NEML** | - NLEM: Basis for outpatient and inpatient reimbursement - Centralised list: for specialised medicines | - Basis for the reimbursement list and hospital medicines lists, does not guarantee state coverage of all listed medicines.^1^ - Permanent Commission of the Ministry of Health to evaluate the NEML, responsible for listing and delisting, based on a Ministry of Health decree |
| **Outpatient** | - For all patients, 100% reimbursement of the defined reimbursement tariff (but co-payments may still exist) | - Revised annually - Based on NEML but can procure high-cost or specialised treatment not on NEML. - Rate of medicines: fully (100%) or partially (50 to 80%) reimbursed following the established fixed amount and available funds). |
| **In-patient** | - Public hospitals must procure medicines included in the NEML can be dispensed - Non-NEML medicines require hospital or patient co-funding | - National Clinical Protocols and NEML are the basis for hospitals to develop their institutional needs for central procurement. - **Criteria**: quality, efficiency, delivery time, payment terms and price. - No co-payment - Revised annually |
| **HTA** | - HTA department established at the State Expert Centre of the MoH (2019) - HTA mandatory for all new medicines | - The HTA process is currently not fully implemented. |

**Table 4 Participant characteristics**

| **Participant** | **Current affiliation** | **Country** |
| --- | --- | --- |
| **1** | International organization | Ukraine |
| **2** | International organization (formally Ministry of Health) | Ukraine |
| **3** | Ministry of Health | Ukraine |
| **4** | International organization | Ukraine |
| **5** | International organization | Ukraine |
| **6** | International organization | Ukraine |
| **7** | International organization (formally Ministry of Health) | Ukraine |
| **8** | International organization | Moldova |
| **9** | International organization (formally Ministry of Health) | Moldova |
| **10** | Ministry of Health | Moldova |
| **11** | Ministry of Health | Moldova |
| **12** | Ministry of Health | Moldova |
